# Development of the DREAM System: Digital Reminders for Everyday Activity Memory

**DOI:** 10.1093/geroni/igab046.736

**Published:** 2021-12-17

**Authors:** Walter Boot, Neil Charness, Sara Czaja, Wendy Rogers, Edie Sanders, Robin Stuart, Ronald Andringa

**Affiliations:** 1 Florida State University, Tallahassee, Florida, United States; 2 Weill Cornell Medicine/Center on Aging and Behavioral Research, New York, New York, United States; 3 University of Illinois Urbana-Champaign, Champaign, Illinois, United States

## Abstract

Prospective memory, the ability to remember to execute an intention in the future, is crucial for the performance of many everyday tasks important for independent living. Prospective memory abilities decline with age, and older adults living with mild cognitive impairment (MCI), cognitive impairment due to traumatic brain injury (TBI), and cognitive impairment due to stroke are especially susceptible to prospective memory failures. The goal of the Digital Reminders for Everyday Activity Memory (DREAM) project is first to establish proof of concept for an adaptive cognitive aid to support the prospective memory of older adults with various cognitive impairments, and then establish proof of product in studies examining the use of a working prototype within the lab and within participants’ homes. Data will be presented from initial work verifying product requirements through engagement with stakeholders, including subject matter experts, older adults with cognitive impairments, and their care partners.

